# Phenolic-Degrading Enzymes: Effect on Haze Active Phenols and Chill Haze in India Pale Ale Beer

**DOI:** 10.3390/foods12010077

**Published:** 2022-12-23

**Authors:** Ilaria Benucci, Caterina Mazzocchi, Claudio Lombardelli, Marco Esti

**Affiliations:** Department of Agriculture and Forest Sciences (DAFNE), Tuscia University, Via S. Camillo de Lellis snc, 01100 Viterbo, Italy

**Keywords:** laccase, tannase, kinetic characterization, haze active phenols removal, chill haze prevention

## Abstract

The development of green and sustainable biotechnological approaches for preventing chill haze formation is currently under investigation. In this preliminary study, laccase and tannase (pure or combined) were applied as phenolic-degrading enzymes during two crucial brewing steps (i. post-mashing and ii. before the yeast inoculum). In post-mashing and irrespective of the dosage applied (100 μL/L or 1 mL/L), tannase-based treatment ensured the complete removal of haze active (HA) phenols, which was proved by the full prevention of chill haze (about 1 EBC vs. 22 EBC in the control sample). Before yeast inoculum for the alcoholic fermentation, the removal of haze active phenols and the prevention of chill haze were both tannase-dosage-dependent (15 and 2 EBC for the lowest and the highest dosages, respectively) although they failed to completely break down the HA phenols. This biotechnological approach did not significantly affect the chromatic properties of treated beer.

## 1. Introduction

Colloidal stability is crucial in beer quality, significantly affecting its shelf life as well as attracting consumers [1,2]. Not all beers are expected to be clear, however haziness has been recognized as a defect for lagers and pilsners [1].

Non-biological haze originates from brewing raw materials (e.g., malted barley and hop) [3,4] due to interactions between haze active (HA) proteins and HA polyphenols, which represent a very small amount of the total beer protein and polyphenol content, respectively [5]. Due to its hydrophobic properties, the amino acid proline represents the primary binding site for polyphenols. Early studies have suggested that barley hordeins are the main source of proline-rich proteins (HA proteins) in beer [6]. It has been proved that phenolic compounds from barley and hops play a crucial role in the formation of beer haze [7]. In particular, proanthocyanidins (oligomeric flavonoids), dimers, and tetramers of catechin, epicatechin, and gallocatechin [5] have been recognized as HA polyphenols. These compounds, which contain two or more binding sites, can form bridges between protein molecules. Nevertheless, the binding mechanism between HA proteins and HA polyphenols is still not clear, probably because of the different simultaneous interactions involved [8].

Chill haze may evolve into permanent haze by a two-stage mechanism. Firstly, non-covalent bonds (e.g., hydrophobic interactions and hydrogen bonds) between HA proteins and HA polyphenols give rise to soluble complexes (chill haze) [3]. These soluble complexes precipitate at low temperatures, forming particles 0.1–1 μm in diameter which rapidly re-dissolve heating beer at temperatures of 20 °C or greater [4]. Permanent haze initially develops similarly to chill haze, but the soluble complexes quickly become insoluble due to the formation of covalent bonds that do not dissolve heating beer [9]. The resulting haze particles increase to colloidal size (1–10 μm in diameter) such that they are able to scatter light and form a permanent visible sediment [4]. 

The conventional approach for the removal of HA polyphenols involves adding a colloidal stabilization agent (e.g., polyvinylpolypyrrolidine-PVPP, gelatin, nylon and lucilite TR) prior to filtration of the beer [1]. The most common way this is accomplished is through adsorption to PVPP. This is because the structural analogy between HA polyphenols and pyrrolidone rings enables them to bind to polymerized flavanoids through hydrogen and ionic bonds. The use of isinglass as a fining agent has also proved to be useful, however its application is limited because it has been declared as an allergen [10,11]. The main concern related to using colloidal stabilization agents is the health hazard associated with the handling of dust before they are applied. Moreover, in light of the increasing need for green processes, the required professional disposal of used materials represents a critical issue. In addition, the filtration step essential for removing the polyphenol–adsorbent complex from beer is energy-consuming, which impairs the treatment’s sustainability. Furthermore, the limited specificity of fining agents may impair beer quality due to the undesirable removal of non-HA polyphenols, some of which play a positive role in beer flavor [12]. 

Purified enzymes obtained from microorganisms have been employed in the beverage industry for decades because of their ability to improve product quality and processes with minimal side effects and low costs [13]. Enzymatic treatment may contribute to the selective removal of HA compounds in beverages [14] as well as HA polyphenols in beer, avoiding an undesirable alteration of their organoleptic characteristics. Thus, it represents a valuable alternative to the conventional approach based on colloidal stabilization agents. 

Laccase (benzenediol:oxygen oxidoreductase; EC 1.10.3.2) is a blue copper oxidoreductase that is able to catalyze both the oxidation of a wide range of substrates (including phenolic compounds) and the simultaneous reduction of molecular oxygen to water [15]. It is used to enhance or modify color appearance in beverages and to increase both the strength and stability and to reduce the stickiness of dough in baking, thereby improving its machinability [16]. Osma et al. [17] described how laccase can be used to control phenolic compounds in beer and wine stabilization. Indeed, laccase is able to prevent haze formation by oxidizing beer phenols.

Tannase (tannin acyl hydrolase; E.C. 3.1.1.20) catalyzes the hydrolysis of ester bonds in gallotannins, complex tannins, and gallic acid esters [18]. Over the years, microbial tannase has been used in the food processing industry as a clarifying agent during the manufacturing of instant tea, beer, fruit juices, and some wines [19]. The high concentration of tannins in these products may result in the formation of precipitates due to their interaction with other molecules. These undesirable effects may be reduced by enzymatic treatment using tannase that could help to release the tannin monomers, thereby enhancing the nutritional properties of such beverages [20].

The brewing process mainly involves nine general steps: malting, milling, mashing, lautering, hoping, fermentation, conditioning, filtering, and canning or bottling. The main enzymes used in the beer brewing industry can be used in four main processes: germination, mashing, fermentation, and clarification [21].

In order to explore the efficacy of two phenolic-degrading enzymes in terms of HA phenol removal and chill haze prevention in beer, laccase and tannase were first characterized in terms of optimal pH and temperature and then used in an India Pale Ale processing procedure. The pure or combined enzymatic activities were applied at two crucial brewing steps (i. post-mashing; ii. after hop flavoring/boiling/cooling and before the yeast inoculum for the alcoholic fermentation), both of which were characterized by the natural enrichment of phenolic compounds from two different sources: malted barley and hop.

## 2. Materials and Methods

### 2.1. Enzymes, Chemicals, and Wort Samples

Laccase (L) from *Aspergillus oryzae* (Novozym^®^ 51003, EC 1.10.3.2, declared activity 1000 U/g) was supplied by Novozymes A/S (Bagsværd, Denmark), whereas the food-grade tannase (T) from *Aspergillus oryzae* (EC 3.1.1.20, declared activity 300 U/g) was from S.I.A.L. srl (Rome, Italy). Syringaldazine, tannic acid, and rhodanine for the enzymatic activity assay; gliadin from wheat; and all other reagents were provided by Merck Life Science srl (Milan, Italy).

The wort samples produced using malt Pilsner (Italmalt S.p.A., Melfi, PZ, Italy) and Cryo Hops^®^ pellets Mosaic (alpha acids 20–24%; added at the dosage of 5 g/L before boiling), were kindly supplied by Free Lions Brewery (Viterbo, Italy) at two different brewing steps of the same production batch: (i) post-mashing (PM) and (ii) after flavoring with Mosaic hop/boiling/cooling and before the yeast inoculum for the alcoholic fermentation (BF). The commercial yeast strain *S. cerevisiae* Nottingham Ale Yeast (Lot#10804590677711V, Lallemand, Verona, Italy) was chosen to conduct the alcoholic fermentation.

### 2.2. Bradford Method

The protein content of both enzymatic preparations was quantified by the Bradford colorimetric assay [22], using BSA as the standard protein.

### 2.3. Laccase Activity Assay

Laccase activity was determined using the revised syringaldazine method [23]. The sample solution, consisting of substrate (1 mM syringaldazine in ethanol 96%), buffer (0.1 M acetate buffer, pH 4.5), and enzyme (diluted 1:400 *v*/*v* in bi-distilled water), was homogenized and the absorbance was read at 530 nm for 3 min in a continuous mode (UV-visible spectrophotometer, Shimadzu UV 2450, Milan, Italy). A blank test (sample that does contain any enzyme) was performed. Assays were carried out in triplicate. Laccase activity was determined from the change in absorbance vs. time using the linear portion of the curve, and it was expressed in IU (ε syringaldazine = 65 mM^−1^ cm^−1^ at 530 nm) [24,25]. 

### 2.4. Tannase Activity Assay

A spectrophotometric method based on the formation of chromogen between gallic acid and rhodanine was applied with some modifications to test tannase activity [26,27]. The sample solution, consisting of substrate (1 mM tannic acid dissolved in 0.1 M acetate buffer, pH 4.5), buffer (0.1 M acetate buffer, pH 4.5), and enzyme (1 mg/mL dissolved in b-idistilled water), was pre-incubated at 20 °C for 5 min. For each trial, a blank, a test, and a control (all containing 5 mL of substrate) were prepared. 5 mL of the buffer were added to the blank and control, whereas 5 mL of the enzyme solution was added to the test. Samples were incubated at 20 °C for 10 min. Aliquots of 0.5 mL were removed every 2 min and 0.3 mL of methanolic rhodanine (0.667% *w*/*v* rhodanine in 100% methanol) was added to each one; the samples were then kept at 30 °C for 5 min. Afterwards, 0.2 mL of KOH (0.5 M) was added and the samples were incubated at 30 °C for 5 min. For the control tube only, this was followed by the addition of enzyme (0.25 mL) to the reaction mixture. Finally, each sample was diluted with distilled water, reaching a final volume of 4.0 mL, and left to stand at 30 °C for 10 min. The absorbance at 520 nm was recorded against water by a UV-visible spectrophotometer (Shimadzu UV 2450, Milan, Italy). Assays were performed in triplicate. A calibration curve over the range 0–1 mM was prepared by using gallic acid. Tannase activity was calculated from the change in absorbance:

ΔABS_520_ = (ABS_test_ − ABS_blank_) − (ABS_control_ − ABS_blank_) (1)

Tannase specific activity was expressed as IU/mg protein (IU/mg_BSAeq_).

### 2.5. Optimal pH and Temperature

To evaluate the effect of pH and temperature on enzyme activity, the assays described in paragraphs 2.3 and 2.4 were conducted using syringaldazine (1 mM) as a substrate for laccase and tannic acid (1 mM) as a substrate for tannase. The pH interference was tested by performing the assays in McIlvaine buffer at a pH of 2.0–8.0, whereas the effect of temperature was evaluated by carrying out the enzymatic assays at the optimal pH at different temperatures ranging from 10 °C to 80 °C.

### 2.6. Enzymatic Treatment of Wort

The effect of different enzyme preparations (consisting of pure or combined enzyme activities) and dosages on the haze active phenols, chill haze, and chromatic properties was evaluated. The wort was treated enzymatically at two different brewing steps of the same production batch: (i) post-mashing (PM) and (ii) after boiling/cooling and before the yeast inoculum for the alcoholic fermentation (BF). For each test, 1 L of sterilized wort was placed in a 1.5-L glass fermentation vessel. The enzymatic treatments were conducted by adding only laccase (L), only tannase (T), or both (L+T) at two different dosages (Table 1). A control test with no enzymes added was also performed. Three biological replicates were prepared for each of the fourteen tests conducted (control and six enzyme preparations for both PM and BF), as summarized in Table 1.

Following the enzymatic treatment, the commercial yeast strain *S. cerevisiae* Nottingham Ale Yeast was inoculated in all samples (pitching rate: 0.8 g/L). The alcoholic fermentation was performed at 20 °C in triplicate and was considered complete when the specific gravity remained constant for 4 days. The efficacy of enzymatic treatment was investigated at different time points following the yeast inoculation (from 24 h to 504 h). Aliquots were collected and centrifuged at 4600 rpm for 5 min (Thermo Fisher Megafuge 16R, Milan, Italy) before the analyses.

### 2.7. Haze Active Phenols

HA phenols were detected according to the process described by Siebert and Lynn [28]. A stock solution of saturated gliadin was prepared by adding excess gliadin to acetate buffer (0.1 M, pH 4.5). The mixture was stirred, sonicated, and left to stand overnight at room temperature; it was then filtered through Whatman No. 2 filter paper. 18 mL of sample (wort or beer) was placed in a beaker to which 2 mL of buffer or 2 mL of the saturated gliadin solution was added. The sample was incubated at 80 °C for 30 min and then at 25 °C for 30 min, after which the turbidity (nephelometric turbidity units, NTU) was measured using a HD 25.2 turbidimeter (Delta Hom, Padova, Italy). The haze difference between the sample with and without added gliadin was reported as the HA polyphenol.

### 2.8. Chill Haze

Colloidal stability was predicted using the alcohol-chill test described by Chapon [29]. After the addition of 6% pure ethanol, samples (9 mL) were incubated for 40 min at −5 °C. The chill haze (expressed as European Brewery Convention units (EBC), based on formazin haze standards) was calculated as the difference between the turbidity (HD 25.2 turbidimeter, Delta Ohm, Padova, Italy) of the sample after and before the cold storage.

### 2.9. CIELAB Parameters and Browning Index

The effect of enzymatic treatment on chromatic properties of samples was determined using a CR-5 colorimeter (Konica Minolta, Tokyo, Japan), a D65 illuminant, and the CIELab* uniform color space. The L* value indicates lightness and darkness, and it ranges from 0 to 100 (0 indicates black; 100 indicates white), whereas the two chromatic components a* (from green to red) and b* (from blue to yellow) range from −120 to 120 [30]. The analyses were performed in triplicate with five measurements in each sample unit. The color changes of samples were evaluated using the total color difference (ΔE):

ΔE = [(ΔL*)^2^ + (Δa*)^2^ + (Δb*)^2^]^1/2^ (2)

Along with this, the browning index (BI) was also determined using the following equation [31]:

(3) $$\mathrm{BI}=\frac{100(x-0.31)}{0.17}$$

(4) $$x=\frac{a*\;+\;1.75\;L*}{5.645\;L*\;+\;a*\;-\;3.012\;b*}$$

### 2.10. Statistical Analysis

All determinations were conducted in triplicate and the results were expressed as mean ± standard deviation. Data were analyzed using one-way analysis of variance (ANOVA) tests using the EXCEL^®^ add-in macro DSAASTAT (Microsoft Excel 2021, Tuscia University, Viterbo, Italy) followed by Tukey Honestly Significant Difference (Tukey HSD) post-hoc tests (α = 0.05) for multiple comparisons of samples.

## 3. Results and Discussion

### 3.1. Laccase and Tannase Activity: Optimal pH and Temperature

**Figure 1:**
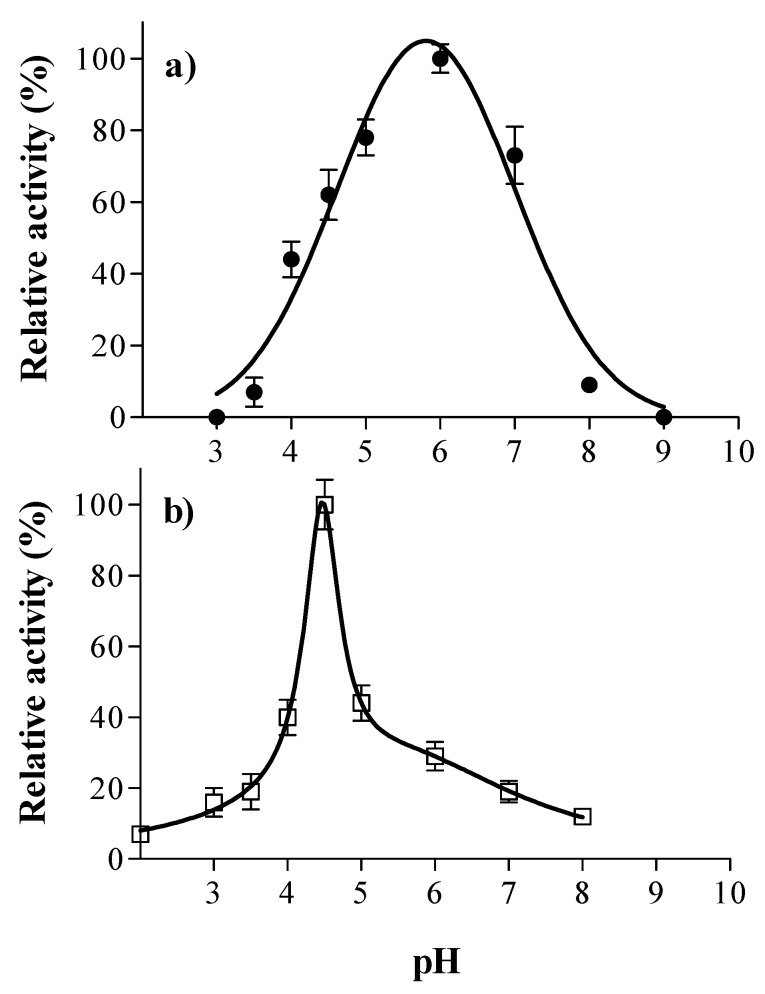
Effect of pH on the activity of laccase (●, (**a**)) and tannase (□, (**b**)) from *A. oryzae*. Assays were performed in McIlvaine buffer using syringaldazine (1 mM) as a substrate for laccase and tannic acid (1 mM) as a substrate for tannase. The points represent the average values, and the error bars represent the standard deviation of the three replicates.

Although the enzymes tested in this study are commercial preparations, they are not exclusively intended for application in beer production. For instance, tannase has several industrial uses such as in the preparation of instantaneous tea or the reduction of tannin levels in fruit juices. Therefore, knowing the optimal pH and temperature of the enzymes can provide valuable information about their relative activity under industrial conditions. Figure 1 shows the activity values for laccase and tannase from *A. oryzae* as a function of pH. 

The results indicated that the optimal pH of laccase is 6 (Figure 1a). About 62% of the relative activity was retained at pH 4.5 (the average pH value of beer), which is within the activity magnitude values observed by Skoronski et al. [32] for the same laccase from *A. oryzae*. It has been reported that most fungal laccases have optimal activity toward phenolic substrates in the pH range 3.0–5.5, becoming inactive at neutral and alkaline pH values [33,34]. In previous studies, the optimal pH was 3.5 for laccase from Botrytis cinerea [35] and 4.5 for laccase from Pleurotus sp [36].

In this study, the optimal pH for tannase activity appeared to be 4.5 (Figure 1b) and was within a narrower range than that of laccase. The enzyme activity increased sharply from pH 4 to 4.5 followed by a rapid decline after 4.5. It has been reported that *A. oryzae* tannase is an acidophilic enzyme with an ideal pH around 5.0 [37]. Mizuno et al. [38] found that the optimal pH for the same purified native enzyme was 4.0. It has been recognized that *A. oryzae* tannase is an acid-stable enzyme and its stability in acidic conditions is advantageous during food processing [38]. The fungal tannases generally have optimal pHs in the acidic range [18,39]. In previous studies, the ideal pH was 6.0 for tannase from Aspergillus niger and Penicillium chrysogenum [39,40] and 5.5 in the case of tannase from Lenzites elegans [41].

**Figure 2:**
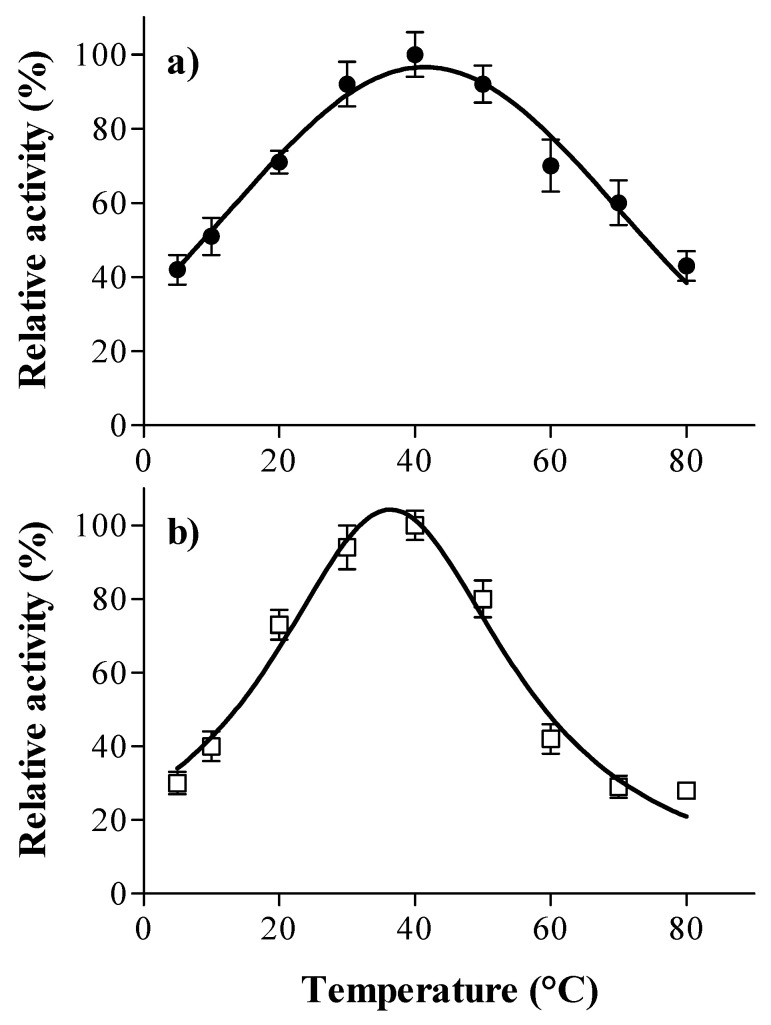
Effect of temperature on the activity of laccase (●, (**a**)) and tannase (□, (**b**)) from *A. oryzae*. Assays were performed in McIlvaine buffer using syringaldazine (1 mM) as a substrate for laccase and tannic acid (1 mM) as a substrate for tannase. The points represent the average values, and the error bars represent the standard deviation of the three replicates.

In this study, the temperature activity profile of laccase and tannase from *A. oryzae* followed a bell-shaped curve (Figure 2). For both enzymes, the optimal temperature was 40 °C. Laccase retained more than 70% of the relative activity in a broad temperature range (20–60 °C) (Figure 2a). It has been reported that the suitable range for fungal laccase activity is from 30 °C to 60 °C. Skoronski et al. [32] found that the highest activity for laccase from *A. oryzae* occurred at at 50 °C, whereas the optimal temperature was 40 °C for laccase from *Botrytis cinerea* [42] and 65 °C for laccase from *Pleurotus* sp. [36].

In this study, Tannase showed a smaller temperature range than that of laccase, maintaining about 70% of the relative activity between 20 °C and 50 °C (Figure 2b). The optimal temperature at 40 °C was similar to that of values reported by Mizuno et al. [38], as well as to those reported for tannase produced by a co-culture of *Rhizopus oryzae* and *Aspergillus foetidus* [43].

### 3.2. Effect of Enzymatic Treatment on the Haze Active Phenols and Haze Stability

The amount and nature of phenolic compounds change throughout the different stages of beer production [44]. Beer phenols from malted barley and hop directly contribute to several characteristics of beer, including haze formation. In order to ascertain the effectiveness of their use as stabilizers in beer processing, laccase and tannase were applied as pure or combined enzymatic activities at different dosages at two crucial brewing steps: i. post-mashing (PM) and ii. after boiling/cooling and before the yeast inoculum for the alcoholic fermentation (BF). The efficacy of enzymatic treatments was investigated at different time points following the yeast inoculation.

#### 3.2.1. The Fate of Haze Active Phenols from Malted Barley and Hop

**Figure 3:**
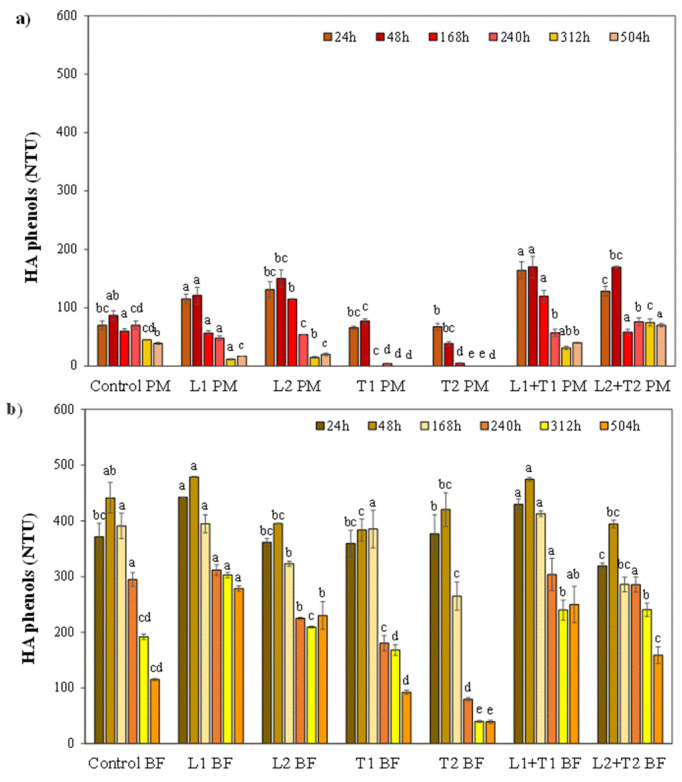
Haze active (HA) phenols (reactive to gliadin) in enzymatically-treated wort (L: Laccase; T: Tannase) at two different brewing steps: (**a**) post-mashing (PM) and (**b**) after boiling/cooling and before the yeast inoculum for the alcoholic fermentation (BF). The efficacy of enzymatic treatments was investigated at different time points following the yeast inoculation (24–504 h). Samples were: control (untreated wort), L1 (100 μL Laccase), L2 (1 mL Laccase), T1 (100 μL Tannase), T2 (1 mL Tannase), L1 + T1 (100 μL Laccasi + 100 μL Tannase), and L2 + T2 (1 mL Laccase + 1 mL Tannase). Reported data are from the triplicate analysis and are presented as mean ± SD. For each time point, data with different letters differ significantly with respect to enzyme treatments. (Tukey’s test, *p* < 0.05).

Mashing is a complex and dynamic phase affected by the synergy of many processes such as the degradation and release of bound phenolics by enzyme action. It usually results in the increase of simple phenolics from malt [45]. As presented in Figure 3a, in PM, the effect of tannase-based treatment at the highest dosage (T2) already appeared to be useful after 48 h when the content of HA phenols (39 NTU) was half that of the control (87 NTU). At the lowest dosage (T1), a significant decrease of HA phenols was revealed after a longer time (72 h, 10 NTU). In both T1 and T2, the reactivity to gliadin was almost undetectable at later time points and the amount of HA phenols was close to zero (Figure 3a). In post-mashing, the application of tannase (especially at the highest dosage) proved to be the most useful treatment, ensuring the complete removal of HA phenols. It may be presumed that tannase catalyzes the hydrolysis of phenolics recognized as HA in malt, including both dimers (prodelphinidin and procyanidin, which are characterized by a higher amount of gallocatechin units than the corresponding compounds in hop) [46] and trimers (catechin and gallocatechin units).

In all the tested samples, the addition of laccase resulted in an initial increase in the reactivity to gliadin (Figure 3a). It might be assumed that the enzymatic preparation contributes to this phenomenon by adding HA substances. Moreover, it is also reasonable that the oxidation of phenolics catalyzed by laccase gives rise to highly reactive oxidized compounds [16]. Irrespective of the dosage, only a slight decrease in HA phenols was detected following laccase treatment, whereas its simultaneous addition with tannase did not significantly contribute to the decrease of HA phenols (Figure 3a).

Data in Figure 3b show that the initial amount of HA phenols in the control BF (about 430 NTU) was remarkably higher compared to the control PM sample (about 80 NTU). This may be partially explained by considering the balance between numerous phenomena, such as direct phenolics extraction from hop [47], water evaporation, depolymerization reactions [48], polyphenol-polyphenol, and protein-polyphenol bounds [45,49,50], which occurs during wort boiling. However, the initial content of HA phenols in the control BF (about 430 NTU) gradually decreased following the yeast inoculation to about 115 NTU, probably due to their sorption to the outer surface of the yeast cell wall. As depicted in Figure 3b, tannase-based treatment was the only treatment useful in breaking down HA phenols in BF. The effect of tannase added at the highest dosage (T2) was appreciable only after 168 h, at which point the content of HA phenols was significantly smaller (265 NTU) than in the control sample (391 NTU). A further decrease was observed at later time points, when the reactivity to gliadin was remarkably diminished (−80% HA phenols, Figure 3b). At the lowest dosage (T1), a significant reduction of HA phenols was detected after a longer time (from 240 h until the end of the trial). In line with the results revealed in post-mashing, the application of tannase at the highest dosage appeared to be the most useful treatment in removing HA phenols. Despite this, the addition of tannase before the yeast inoculum for the alcoholic fermentation failed to provide a complete breakdown of HA phenols. It is reasonable to suppose that this result may be due to the high content of flavonoid oligomers and procyanidin dimers and trimers derived from hop [47] as well as to the presence of more complex compounds (reactive to gliadin) prone to produce haze which tannase is unable to act on. All the other enzymatic treatments conducted before the alcoholic fermentation (i.e., adding only laccase or laccase combined with tannase), did not significantly contribute to the decrease of HA phenols (Figure 3b).

#### 3.2.2. Chill Haze Behavior

**Figure 4:**
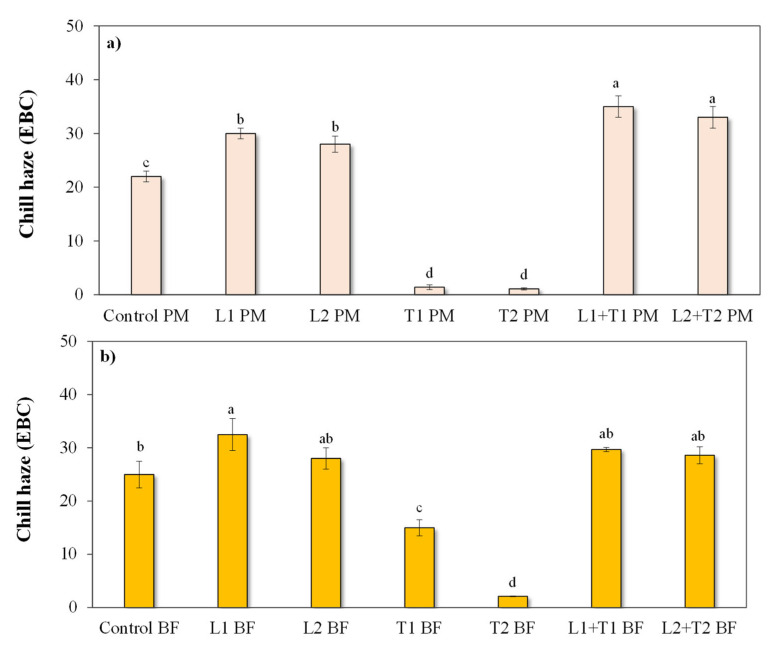
Chill haze (EBC) of enzymatically-treated wort (L: Laccase; T: Tannase) at two different brewing steps: (**a**) post-mashing (PM) and (**b**) after boiling/cooling and before the yeast inoculum for the alcoholic fermentation (BF). Data refer to the chill haze 504 h after the yeast inoculation. Samples were: control (untreated wort), L1 (100 μL Laccase), L2 (1 mL Laccase), T1 (100 μL Tannase), T2 (1 mL Tannase), L1 + T1 (100 μL Laccasi + 100 μL Tannase), and L2 + T2 (1 mL Laccase + 1 mL Tannase). Reported data are from the triplicate analysis of wort and are presented as mean ± SD. Data with different letters differ significantly with respect to enzyme treatments. (Tukey’s test, *p* < 0.05).

The effectiveness of enzymatic treatments on chill haze depletion 504 h after the yeast inoculation is presented in Figure 4. In both PM and BF, a remarkable decrease in chill haze was only revealed in samples treated with tannase, thereby proving that the loss in HA phenols is widely significant in relation to chill haze prevention.

However, some turbidity does not stem from polyphenol hazing, but rather is permanent haze from other sources (e.g., yeast cells). In line with the above results, data in Figure 4a provide further evidence that in PM, the application of tannase (at both dosages) resulted in almost a complete absence of chill haze (about 1 EBC in T1 PM and T2 PM vs. 22 EBC in the control PM) due to the selective hydrolysis of HA phenols involved in haze formation, as previously discussed. A significant decrease of chill haze was also revealed in BF and was associated with a tannase-dosage-dependent effect (15 and 2 EBC in T1 BF and T2 BF, respectively). These results confirm that the greater the degree of HA phenol removal, the greater the chill haze depletion, as previously observed for tannase-based treatment in BF. Bearing in mind that a linear correlation between tannins and haze formation does not exist, a linear relationship for the haze magnitude and a polyphenol threshold concentration could be described for the haze formation. Neither the use of laccase nor its simultaneous application with tannase significantly contributed to the prevention of chill haze (Figure 4a,b).

### 3.3. Effect of Enzymatic Treatment on Color

Beer color is an important sensory attribute, and is the first one that the consumer experiences. The color of the final product is mainly a result of the different raw materials used during the brewing process and by the oxidation of polyphenols originating from malted barley and hops. L*, a*, and b* coordinates and ΔE* color differences may be used to objectively estimate the color difference between two samples. In detail, ΔE* < 1.5 indicates almost no difference upon visual inspection, 1.5 < ΔE* < 3 suggests a slight difference, 3 < ΔE*< 6 indicates there are some significant differences, and ΔE* > 6.0 means that the colors of the samples are totally different [30]. Considering the values in Table 2, it is clear that the samples treated with tannase had chromatic characteristics more similar to the sample in which the enzyme was not added (control), regardless of the step of the process being considered (PM or BF).

ΔE* values indicated only a partial difference of the samples (ΔE* = 2.9 for T2PM) and no color difference was evident for the T1 BF and T2 BF samples (ΔE* < 1.5). The browning index (BI) remained almost the same as in the untreated sample, with no significant variations (Table 2). These observations agree with what was reported by Eissa et al. [51] who investigated the use of tannase in grape juice clarification and found no appreciable changes in the CIElab and BI indices. Compared to the tannase-treated samples, the samples treated with laccase showed a much greater effect on the chromatic properties in both the PM and BF samples. In particular, the ΔE* values indicated a difference in the color of the treated samples compared to the Control (ΔE* > 6). This trend was also observed in samples treated with the enzyme mix (L + T). The BI of samples treated with laccase (as pure or combined activity) showed the highest values, which is in accordance with the findings of Minussi et al. [52] who reported that treatments with laccase increased the susceptibility to browning in both fruit juices and beer.

## 4. Conclusions

In this study, laccase and a food-grade tannase, both from *Aspergillus oryzae*, were applied as pure or combined enzymatic activities for the removal of HA phenols and the prevention of chill haze in beer. The optimal conditions for tannase activity were closer to the average parameters of the brewing process, whereas laccase retained about 62% of the relative activity at the average pH value of beer. Among treatments, those that used tannase as a pure enzyme were the most suitable in terms of HA phenol removal and chill haze prevention in both brewing steps tested (post-mashing and before the yeast inoculum for the alcoholic fermentation). In post-mashing and at both tested concentrations, tannase-based treatment allowed for the complete and selective hydrolysis of HA phenols involved in haze formation. Before the yeast inoculum, both the reduction of HA phenol reactivity and the prevention of chill haze were tannase-dosage-dependent. The usefulness of laccase was very limited as it was only found in the oxidation of a small amount of HA phenols in post-mashing without a corresponding relevant chill haze prevention.

## Figures and Tables

**Table 1:** Experimental design: treatment groups.

Sample Code	Enzyme Dosage (V_E_/L)
Control PM	-
L1 PM	100 μL Laccase/L
L2 PM	1 mL Laccase/L
T1 PM	100 μL Tannase/L
T2 PM	1 mL Tannase/L
L1 + T1 PM	100 μL Laccase + 100 μL Tannase/L
L2 + T2 PM	1 mL Laccase + 1 mL Tannase/L
Control BF	-
L1 BF	100 μL Laccase/L
L2 BF	1 mL Laccase/L
T1 BF	100 μL Tannase/L
T2 BF	1 mL Tannase/L
L1 + T1 BF	100 μL Laccase + 100 μL Tannase/L
L2 + T2 BF	1 mL Laccase + 1 mL Tannase/L

V_E_: volume of enzyme (μL or mL); PM: wort post-mashing; BF: wort after hop flavoring/boiling/cooling (before alcoholic fermentation); L: Laccase; T: Tannase.

**Table 2:** Effect of different enzyme preparations (pure or combined enzyme activities) and dosages on the color characteristics (L*, a*, b*, and ΔE*) and browning index (BI) of beer.

Sample	L*	a*	b*	ΔE	BI
Control PM	83.5 ± 0.1 ^a^	4.2 ± 0.1 ^b^	18.7 ± 0.1 ^c^	0.00	28.7 ± 0.2
L1 PM	80.2 ± 0.1 ^ab^	4.3 ± 0.1 ^b^	22.0 ± 0.1 ^b^	6.7 ± 0.1	35.4 ± 0.1
L2 PM	77.4 ± 0.1 ^b^	7.6 ± 0.1 ^a^	31.0 ± 0.1 ^a^	14.2 ± 0.1	57.4 ± 0.2
T1 PM	87.2 ± 0.1 ^a^	3.2 ± 0.1 ^b^	14.7 ± 0.1 ^c^	5.6 ± 0.1	20.8 ± 0.1
T2 PM	85.4 ± 0.1 ^a^	2.0 ± 0.1 ^b^	19.1 ± 0.01 ^c^	2.9 ± 0.1	26.6 ± 0.1
L1 + T1 PM	80.6 ± 0.1 ^a^	6.3 ± 0.1 ^a^	20.6 ± 0.4 ^c^	6.1 ± 0.2	34.9 ± 0.1
L2 + T2 PM	81.7 ± 0.1 ^a^	6.2 ± 0.1 ^a^	27.3 ± 0.1 ^a^	8.9 ± 0.1	45.4 ± 0.1
Control BF	93.5 ± 0.1 ^a^	−1.2 ± 0.1 ^c^	29.6 ± 0.1 ^b^	0.00	36.1 ± 0.1
L1 BF	86.4 ± 0.1 ^b^	4.5 ± 0.1 ^b^	37.7 ± 0.1 ^a^	12.2 ± 0.1	59.5 ± 0.1
L2 BF	83.2 ± 0.1 ^b^	6.4 ± 0.1 ^a^	39.6 ± 0.1 ^a^	16.3 ± 0.1	68.3 ± 0.1
T1 BF	93.3 ± 0.1 ^a^	−1.3 ± 0.1 ^c^	29.8 ± 0.1 ^b^	0.3 ± 0.1	36.4 ± 0.2
T2 BF	93.7 ± 0.1 ^a^	−1.1 ± 0.1 ^c^	30.0 ± 0.1 ^b^	0.4 ± 0.1	36.6 ± 0.1
L1 + T1 BF	87.9 ± 0.1 ^b^	3.4 ± 0.1 ^b^	35.4 ± 0.1 ^a^	9.3 ± 0.1	53.2 ± 0.7
L2 + T2 BF	84.0 ± 0.1 ^b^	6.4 ± 0.1 ^a^	40.3 ± 0.1 ^a^	16.1 ± 0.1	68.8 ± 0.1

Samples were: control (untreated sample), L1 (100 μL Laccase), L2 (1 mL Laccase), T1 (100 μL Tannase), T2 (1 mL Tannase), L1 + T1 (100 μL Laccasi + 100 μL Tannase), and L2 + T2 (1 mL Laccase + 1 mL Tannase). Reported data are from the triplicate analysis of beer and are presented as mean ± SD. Data with different letters differ significantly with respect to enzyme treatments. (Tukey’s test, *p* < 0.05). PM: wort post-mashing; BF: wort after hop flavoring/boiling/cooling (before alcoholic fermentation); L: Laccase; T: Tannase.

## Data Availability

All related data and methods are presented in this paper. Additional inquiries should be addressed to the corresponding author.
